# Diabetic encephalopathy: beneficial effects of supplementation with fatty acids ω3 and nordihydroguaiaretic acid in a spontaneous diabetes rat model

**DOI:** 10.1186/s12944-018-0938-7

**Published:** 2019-02-08

**Authors:** Gustavo Tomás Díaz-Gerevini, Alejandro Daín, María Eugenia Pasqualini, Cristina B. López, Aldo R. Eynard, Gastón Repossi

**Affiliations:** 1 Biología Celular, Histología y Embriología. Facultad de Ciencias Médicas, INICSA CONICET-Universidad Nacional de Córdoba, Córdoba, Argentina; 2 Geriatric Center “San Ricardo Pampuri”, Villa Carlos Paz and Gerontology Committee, Argentine Society of Diabetes, Córdoba, Argentina; 3 Cátedra de Histología y Embriología, Universidad Nacional de La Rioja (UNLaR), La Rioja, Argentina

**Keywords:** Diabetic encephalopathy, Polyunsaturated fatty acids ω3, Nordihydroguaiaretic acid, eSS rats, diabetes mellitus

## Abstract

**Background:**

Diabetic encephalopathy is a chronic complications of diabetes mellitus that affects the central nervous system. We evaluated the effect of ω3 and ω6 polyunsaturated fatty acids (PUFAs) supplementation plus the antioxidant agent nordihydroguaiaretic acid (NDGA) on the etiopathology of diabetic encephalopathy in eSS rats, a spontaneous model of type 2 diabetes.

**Methods:**

One hundred twenty spontaneous diabetic eSS male rats and 38 non-diabetic Wistar, used as healthy control, received monthly by intraperitoneal route, ω3 or ω6 PUFA (6.25 mg/kg) alone or plus NDGA (1.19 mg/kg) for 12 months. Diabetic rats had a worse performance in behavioural Hole-Board test. Histopathological analysis confirmed lesions in diabetic rats brain tissues. We also detected low expression of synaptophysin, a protein linked to release of neurotransmitters, by immunohistochemically techniques in eSS rats brain. Biochemical and histopathological studies of brain were performed at 12th month. Biochemical analysis showed altered parameters related to metabolism. High levels of markers of oxidative stress and inflammation were detected in plasma and brain tissues. Data were analysed by ANOVA test and paired t test was used by comparison of measurements of the same parameter at different times.

**Results:**

The data obtained in this work showed that behavioural, biochemical and morphological alterations observed in eSS rats are compatible with previously reported indices in diabetic encephalopathy and are associated with increased glucolipotoxicity, chronic low-grade inflammation and oxidative stress burden. Experimental treatments assayed modulated the values of studied parameters.

**Conclusions:**

The treatments tested with ω3 or ω3 plus NDGA showed improvement in the values of the studied parameters in eSS diabetic rats. These observations may form the basis to help in prevent and manage the diabetic encephalopathy.

**Electronic supplementary material:**

The online version of this article (10.1186/s12944-018-0938-7) contains supplementary material, which is available to authorized users.

## Introduction

Diabetic encephalopathy (DE) is a chronic complication of diabetes mellitus that affects the central nervous system (CNS) and is characterized by cognitive impairment and motor dysfunctions that can cause postural balance impairment. The physiopathology of DE could be attributed to long-standing hyperglycaemia, elevated blood pressure, hyperinsulinemia, frequent and severe episodes of hypoglycaemia, and dyslipidaemia.

There is evidence linking type 2 diabetes mellitus (DM2) with low grade chronic inflammation (LGCI) [1, 2]. Hence, in a murine model of spontaneous DM2, the Stillman-Salgado (eSS) rats, we studied possible association among DE [3], neurocognitive alterations and glicolipotoxicity [4].

The concept of glucolipotoxicity refers to the combined, deleterious effects of elevated glucose, triglycerides (TG), higher energy intake and free fatty acid levels (FFA) on pancreatic beta-cell function and survival. Excessive levels of circulating FFA and glucose leads to decreased insulin secretion, impaired insulin gene expression, and in turn beta-cell death by apoptosis [4, 5]. Several pathways have been implicated in fatty-acid inhibition of insulin gene expression, mainly by the extracellular-regulated kinase (ERK1/2) pathway, the metabolic sensor Per-Arnt-Sim kinase and the ATF6 branch of the unfolded protein response [4].

Increased lipid storage in non-adipose tissues may appear in the setting of high levels of plasma FFA or triglycerides (TG) that could lead to “lipotoxicity”. Studies performed in experimental animals and humans suggested that lipotoxicity may occur due to altered energy balance as it happens in DM2, neurodegenerative diseases such as Parkinson’s disease, Alzheimer’s (AD), amyotrophic lateral sclerosis, and heart failure [3, 5, 6]. Accumulation of lipids in heart, skeletal muscle, pancreas and liver tissues may play an important role in the pathogenesis of these diseases [7].

Plasma concentrations of FFA are elevated in the obese subjects and in those with metabolic syndrome. These elevated FFA and non-esterified FFA levels can induce lipotoxicity, due to oxidative stress, which may impair insulin signalling and glucose response in pancreatic β-cells [4]. Experimental and clinical data suggest that saturated FFA such as palmitic acid (PA) which are present in red meat, plays a critical role in the inhibition of the insulin signalling pathway and induction of endoplasmic reticulum (ER) stress in several tissues including hypothalamic neurons. It is likely that ER stress in hypothalamic neurons may lead to AD-like pathological abnormality in primary cortical neurons. Elevated oxidative stress and FFA metabolism when it occurs in astrocytes, it may lead to an increase in their apoptotic cell death, PC12 cells and neural progenitor cells [6]. These chronic metabolic injuries on the central nervous system (CNS) in DM2, in the long run, may result in cognitive impairment and motor dysfunctions which can result in the onset of DE [3].

Epidemiological, clinical and experimental evidences revealed that mild type DM2 may result in subtle and progressive metabolic abnormalities and slow but definite onset of cognitive dysfunction especially in the presence of an imbalance between PUFAs of families ω6 and ω3 (ω6/ω3) [8].

Based on these evidences, we evaluated the effects of intraperitoneal administration of PUFAs ω6 and ω3 alone or in combination with nordihydroguaiaretic acid (NDGA), a potent natural antioxidant compound isolated from native plant *Larrea sp*. [9], on the etiopathology of glucolipotoxicity observed in DE and focused on hippocampal tissues in a spontaneous rat DM2 model.

## Materials and methods

### Ethical disclosure

All animal procedures were approved by the local animal care committee (CICUAL, Facultad de Ciencias Médicas, Universidad Nacional de Córdoba, Argentina) and were developed according to international regulations.

### Animals

eSS rats are a stable variety derived from Wistar strain characterized by Tarres and colleagues [10, 11]. eSS rats develop hypertriglyceridemia around 4th and DM2 at 6th months of age, which is more evident in males [10, 11]. The study was carried out with 158 male rats: 120 diabetic eSS and 38 non-diabetic Wistar rats that formed the healthy control. Animals were kept at 22 ± 2 °C temperature, 65 ± 10% relative humidity, and 12 h light/dark cycle. Rats were provided with commercial chow pellets (GEPSA, Grupo Pilar S.A.) and water ad libitum in the animal facilities of the Instituto de Investigaciones en Ciencias de la Salud (INICSA), CONICET-UNC, Córdoba, Argentina. Efforts were made to minimize animal suffering and to reduce the numbers of animals used.

### Reagents

Arachidonic Acid (AA), nordihydroguaiaretic acid (NDGA) and Xylenol Orange tetrasodium salt were purchased from Sigma-Aldrich® (USA). Fish oil containing 35% eicosapentaenoic acid (EPA) and 40% docosahexaenoic acid (DHA) from Natufarma Labs (Buenos Aires, Argentina). Blood glucose and glycated haemoglobin (HbA1c) were determined by rapid test Accu-check by Roche (Switzerland). Total cholesterol (Chol), TG, C-Reactive Protein (CRP), enzymes: transaminases, pyruvic oxaloacetic, glutamic oxaloacetic and gamma glutamyltranspeptidase (GGTP) diagnostic kits were purchased from Wiener Lab® (Argentina). Rabbit polyclonal anti-synaptophysin (SYN) antibody was purchased from Dako Biotechnology (Denmark). IL-6 plasma levels were measured by immuno-ELISA OptEIA™ system and apoptosis determined by Flow Cytometry (Annexin V: PE) both detection kits were provided by BD Biosciences® (USA). Fatty acids methyl ester (FAME) were identified using a commercial standard Nu-check® (USA) [12]. Hippocampal IL-6 levels were determined by RT-PCR, supplies was purchased from Life Technologies (USA). Apoptotic cells in histological slides were identified by TunelS7100 ApopTag® Peroxidase in situ Apoptosis Detection Kit (Germany).

### Experimental design

All animals received once in a month for 12 months 0.4 ml of treatment by IP route. In these trials the effects of using low concentrations, but ensuring a high absorption, for a long time were studied. These concentrations also decrease the risk of adverse effects. This form of administration and doses has already been tested and published previously in murine models of metabolic syndrome and diabetes [9, 13]. The experimental animals were subjected to the treatments detailed in Table 1.

**Table 1:** Experimental groups and content of IP injections received as treatments

Rat strain	Group name	Treatment (0.4 ml IP)
eSS	Control eSS	Diabetic control. Only saline isotonic solution
eSS	ω6	Saline isotonic solution plus 6.25 mg/kg of 90% AA
eSS	ω6 + NDGA	Saline isotonic solution plus 6.25 mg/kg of 90% AA oil + 1.19 mg/kg NDGA
eSS	ω3	Saline isotonic solution plus 6.25 mg/kg of commercial fish oil containing 35% eicosapentaenoic acid (EPA) and 40% docosahexaenoic acid (DHA)
eSS	ω3 + NDGA	Saline isotonic solution plus 6.25 mg/kg of commercial fish oil + 1.19 mg/kg NDGA
Wistar	Wistar	Non- diabetic control. Only saline isotonic solution

### Tissue sampling

Venous blood from fasting animals, which were slightly anesthetized with isofluorane, was taken from tail vein and centrifuged for 10 min at 1000x g. Plasma obtained was used for measuring various metabolic parameters: blood glucose levels, HbA1c, TGs and Cholesterol at 2, 6 and 12 months. Oxidative stress (GGTP and peroxides), LGCI markers (CRP and IL-6) and total fatty acid (FA) profile were determined in the plasma of blood obtained by cardiac puncture at the time of euthanasia.

Euthanasia was performed by overdose of isofluorane (2,5 ml/kg) and blood obtained at this time by cardiac puncture was heparinized and centrifuged for 10 min at 1.000×g. Brain was excised and one half was fixed in 10% formaldehyde, embedded in paraffin and 6 μm slices were obtained. The other half was frozen at − 80 °C for further studies.

Histopathological analysis and determination of apoptosis, IL-6 and SYN were carried out in the dissected hippocampal tissues.

The following parameters were evaluated in our study:

### Weight

All the animals were examined clinically and weighted monthly under standard conditions (see summary of weight curves in Additional file 1).

### Behavioural testing

Tests were carried out at the end of 6th and 12th month of age. Assays were conducted under day light between 09:00 and 11:00 a.m. after fasting overnight from 19:00 onwards. Equipment was maintained in the same position and temperature on each occasion. The Hole-Board test box consisted of a behavioral assay which provides three major patterns of behavior that broadly can be named as: exploration (in motivational terms), neophilia and neophobia (see Additional file 2). Neophilia is defined as the attraction that animals display towards a moving object or place, while neophobia is aversion that animals show towards an approaching and unknown object or place [14]. The device used in the present study was a modification from the one previously tested [15] consisting of a box (50 × 50 cm), with walls and floor painted white. Four holes (2.5 cm diameter) were placed in two lines in the floor, equidistant from each other and from the walls. Each rat was placed in the same corner. During 10-min length, the following behavioral criteria were recorded by the same observer: (i) moving time period in wandering; (ii) head-dip: the animal places its head into one of the holes, to a minimum depth such that the ears were at the same level of the floor of the box (an additional bout of head-dip was recorded if the animal raised its head fully out of the hole before resuming) [14–16]; (iii) rear: the animal remains stationary on its back legs and raises its fore arms off the ground, extending its body vertically in bipedestation [17].

### Computed Tomography (CT) imaging

Brain CT images were obtained in four anaesthetized rats from each group, with a Picker scanner. Images were analyzed with the Fiji ImageJ v1.51 software (USA) (see Additional file 3) [18].

### Profile of total plasmatic FA by gas chromatography (GC)

Plasma lipids were extracted by Folch’s method and methylated with sodium methoxide. The quantification and identification of total FA methyl ester resultants were performed using a capillary column (BPX 20 m longitude, 0.25 mm ID, 0.25 μm film, SUPELCO©, USA) in a Clarus500© (Perkin-Elmer®) GC and all fatty acids were identified using a commercial standard (Nu-check, USA®) [12].

### Histopathological analysis

Not less than five brains were fixed in each group, processed, cut and sections stained with hematoxylin-eosin (HE) or Nissl. Hippocampal CA1 zone were evaluated by optical microscopy at 10x and 40x magnification in two slides by brain with 4 non-serial 6 μm thick sections each (*n* = 8 sections per animal, 40 per group). To characterize the morphological changes in hippocampus, the thickness of neurons layer and cell dispersion in CA1 zone, were recorded by two examiners blinded to the condition and treatment of each rat brain. The measurements were made with the Fiji ImageJ software.

### Hippocampus cell isolation

Under lens, hippocampi were removed, cut into small pieces (1–2 mm3), immersed in 0.3 ml of 0.25% trypsin solution and incubated at 37 °C for 30 min. The resulting cell suspensions were washed and filtered sequentially through 40 μm cell filters (BD Falcon®) and resuspended in PBS and thus, the number of cells was counted. These cells were used by apoptosis detection in flow cytometer.

### Apoptosis

Apoptosis levels were determined in hippocampal isolated cells of experimental animals by Flow Cytometry using an Annexin V: PE detection kit. Apoptotic cells were also detected in histological preparations by the TUNEL technique.

### LGCI markers

High-sensitive CRP was determined by immunoturbidimetry method with latex particles. Plasmatic IL-6 levels were detected by immunoassay and quantified by means of calibration curve using a commercial standard.

In hippocampus cells IL-6 mRNA expression was assayed by PCR-RT. Total RNA was extracted with TRIzol ® (Life Technologies) and was quantified using a fluorometric method (The Qubit ® 2.0 Fluorometer, Life Technologies). Samples of DNA copy was amplified in triplicate with a StepOnePlus RT-PCR System (Applied Biosystems). For each sample, the amount of mRNA was normalized against the bulk of mRNA of β-actin (used as reference gene). Oligonucleotide sequence was:

IL6_FwTAGTCCTTCCTACCCCAACTTCC,

IL6_RevTTGGTCCTTAGCCACTCCTTC;

β-actin_FwCCGCATCCTCTTCCTCCCT,

β-actin_RevGCCACAGGATTCCATACCCAG [19].

### Oxidative stress biomarkers

GGTP specific activity was determined spectrophotometrically at 405 nm in plasma and brain tissue by a commercial kit (Wiener Labs, Argentina). Peroxides levels were measured in plasma by xylenol orange method [20].

### Hepatic function

As a way to determine gross perturbations in the physiology of liver, plasmatic levels of hepatic enzymes were evaluated.

### Statistical analysis

The data obtained from experiments were analyzed by ANOVA test for the comparison of means. Measurements of the same parameter at different experimental times were analyzed by paired t test. A value of less than 0.05 (*p* < 0.05) was used to define significant differences in all tests. Statistical tests were performed using the InfoStat 3.1 software (Grupo InfoStat, FCA, Universidad Nacional de Córdoba, Argentina).

## Results

### Weight

The eSS rats showed a greater increase in their body weight in the first month compared to Wistar group, but all groups reached a similar weight by the end of the study period. As expected in this model no obesity was observed (see Additional file 1).

### Glycaemia and Oral glucose tolerance test (OGTT)

**Fig. 1:**
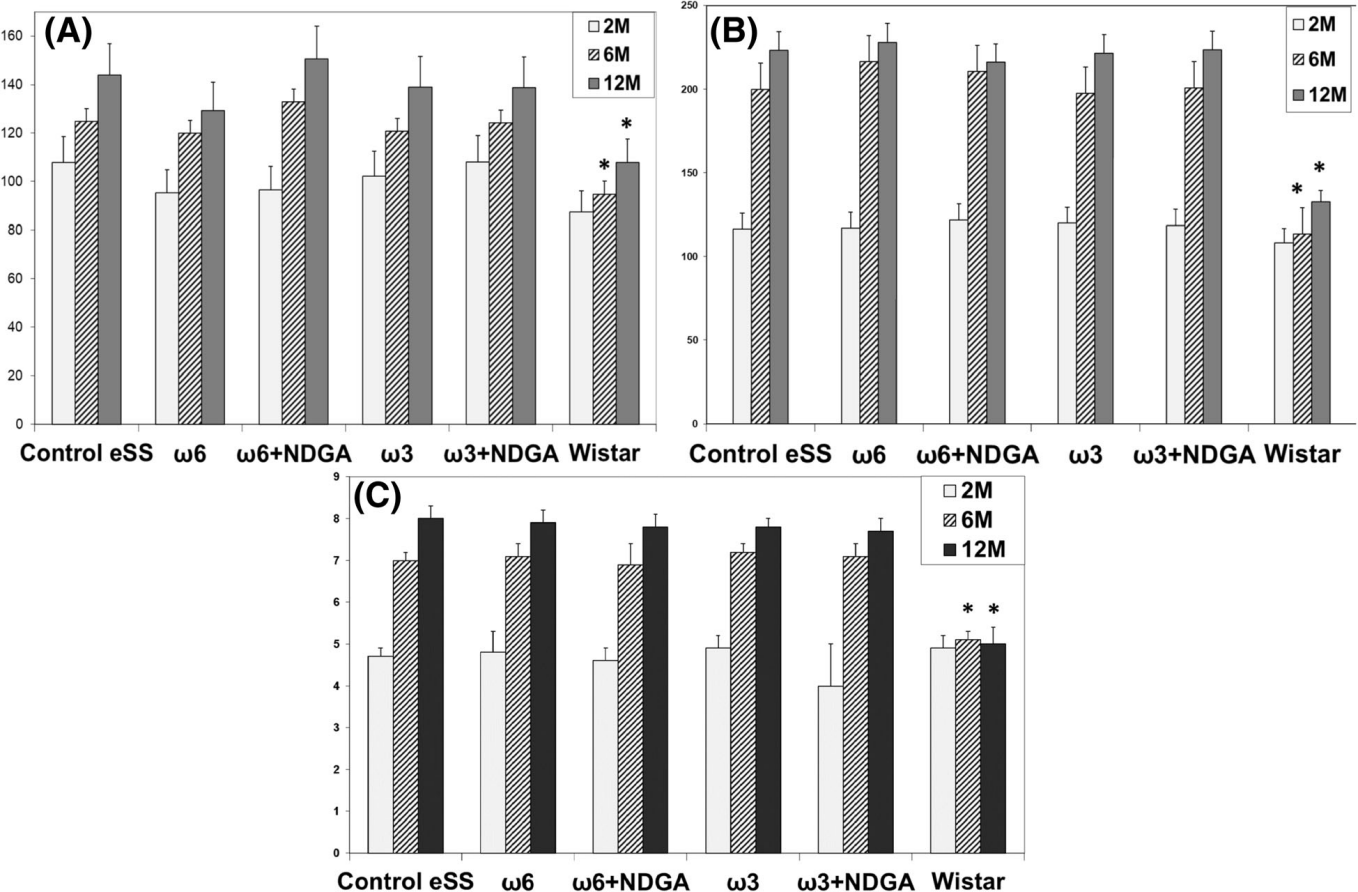
Glycaemic metabolism. OGGT of experimental animals performed at 2, 6 and 12 months. **a** Fasted glycaemia (mg/dl), **b** postprandial glycaemia (mg/dl) and **c** HbA1c levels (%). Data are expressed as mean ± SEM. * Indicate significant difference to Control eSS, *p* ≤ 0.05

All eSS rats developed diabetes within 6th month of life (120/120: 100%). There were no differences in the plasma glucose levels among diabetic eSS groups. Wistar group maintained normal values (Fig. 1a and b).

### HBA1c

In all eSS rats reaching the 6th month of age, HbA1c levels were abnormal i.e. > 6 mg/dl, without any significant difference among all treatments studied. On the other hand, HbA1C remained normal (≤6 mg/dl) in Wistar rats (Fig. 1c).

### Triglyceridemia

**Fig. 2:**
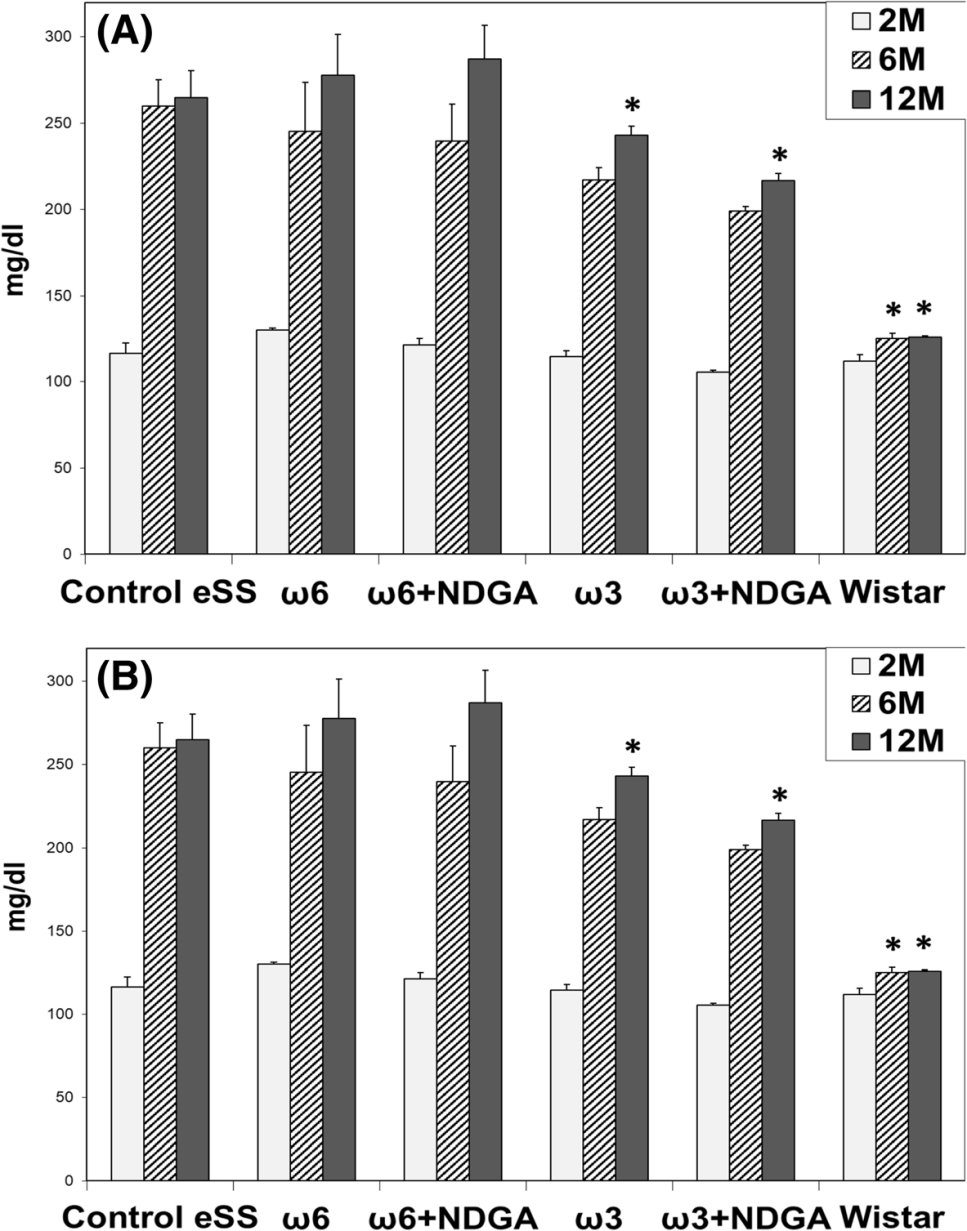
Blood lipids. Levels of plasmatic (**a**) triglycerides and **b** total cholesterol of animals at 2, 6 and 12 months. Data are expressed as mean ± SEM. * Indicate significant difference to Control eSS, *p* ≤ 0.05

In groups of eSS rats, TG showed normal values at 2 months of age. But by 6th month of age TGs were abnormal that remained high even at 12th month of age. Even though ω3 + NDGA treatment induced a decrease in TG values, these values were not statistically different from the Control eSS. TG in Wistar group continued within normal range (Fig. 2a).

### Cholesterolemia

Cholesterol values persisted within the normal range in all groups (Fig. 2b).

### Plasmatic profiles of total fatty acids

Total FA profiles in plasma at 12th month of age showed variations among treatment groups. As shows the Table 2, results grouped in a summarized form according to their degree of unsaturation, showed significant differences that could be related to the type of PUFAs administered. It is evident from these results that administration of ω6 and ω3 PUFAs produced an increase in their respective total PUFAs, in the groups that received them. NDGA addition produced an increase in PUFAs levels (Table 2).

**Table 2:** Plasma total fatty acid profiles by GC

Experimental groups												
	Control eSS		ω6		ω6 + NDGA		ω3		ω3 + NDGA		Wistar	
Fatty acids												
SFA	23,56^#^	±2,28	45,89^#*^	±3,46	38,20^#*^	±3,18	22,20^#*^	±0,34	25,00^#*^	±2,90	27,90^*^	±2,53
MUFAs	27,60^#^	±2,60	17,46^#*^	±1,51	24,50^#*^	±1,29	33,30^*^	±0,25	45,40^#^	±0,76	22,50^*^	±1,36
PUFAs ω6	38,20	±3,65	30,37^#*^	±0,10	23,70^#*^	±1,09	29,00^*^	±0,04	12,00^*^	±0,44	38,00^*^	±3,18
PUFAs ω3	4,00^#^	±0,54	1,83	±0,01	6,20	±0,33	15,10	±0,16	19,30^#^	±0,48	16,40^*^	±0,36
PUFAs Total	42,20	±2,09	32,20^#^	±0,05	29,10^#^	±0,71	44,00	±0,10	31,10^#^	±0,46	54,30^*^	±1,77
Ratio ω6/ω3	9,55^#^		16,60^#^		3,82^#*^		1,92^*^		0,62^*^		2,32^*^	

Profiles was determined at 12TH month of age in experimental groups

^#^Indicate significant difference to Wistar, *p* ≤ 0.05. ^*^Indicate significant difference to Control eSS, *p* ≤ 0.05

### Cognitive-behavioural data: Hole-board test

**Fig. 3:**
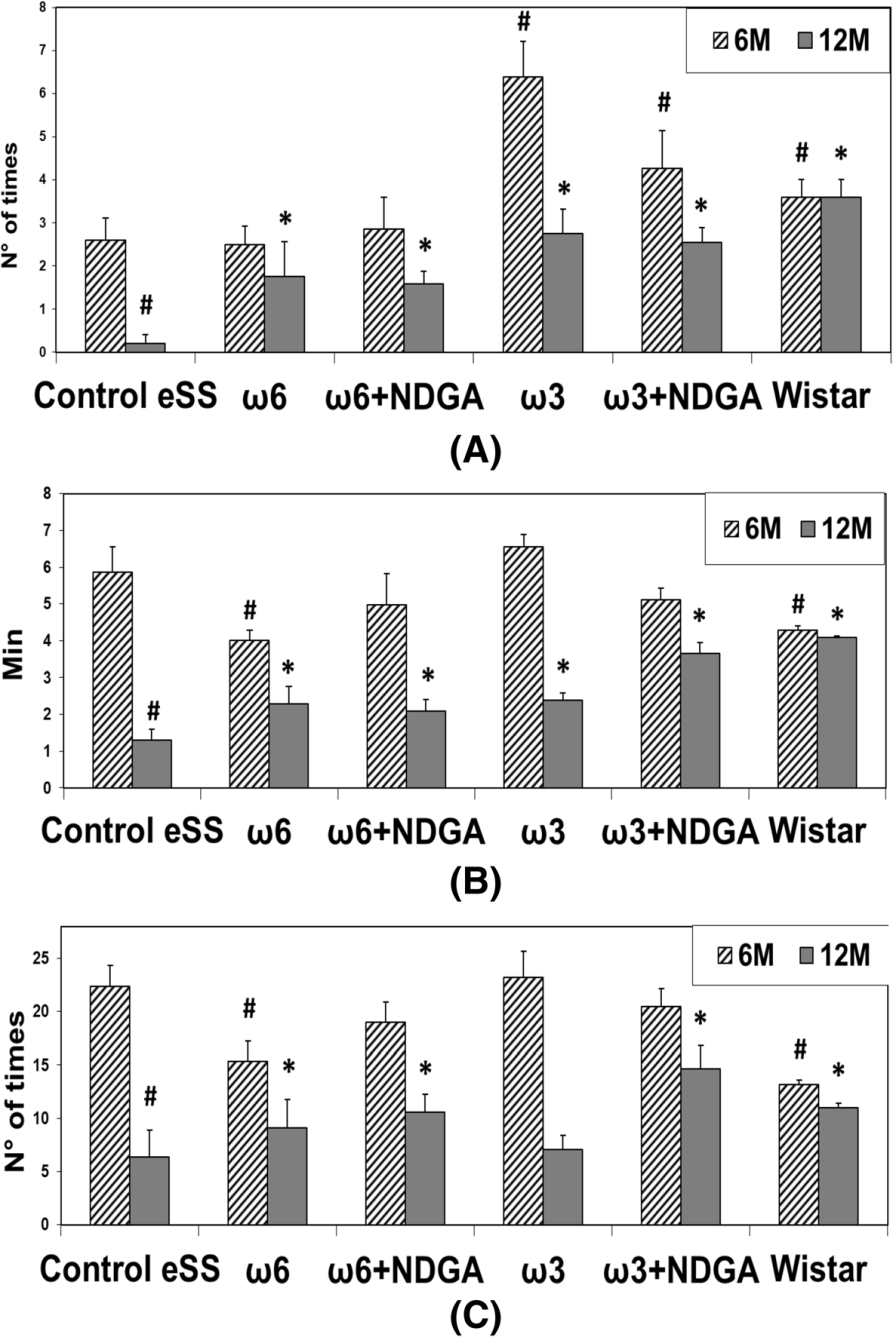
Behaviour Evaluation by Hole Board Test. **a** Head deeping, **b** Time Movement and **c** Rearing. Data are expressed as mean ± SEM. # Indicate significant difference to Control eSS at 6th month, *p* ≤ 0.05. * Indicate significant difference compared to Control eSS at 12th month, *p* ≤ 0.05

At the end of the 6th month of the study, eSS rats were significantly more active in the behavioural parameters compared to non-diabetic rats (Fig. 3). When the accomplishments of tasks were compared, total records of locomotion, frequency of rearing, and head-dip at the end of 12th month, they were significantly worse in untreated diabetics rats. These parameters showed improvement at the end of 12th month in those which were administered ω3 alone or ω3 + NDGA. On the contrary, rats treated with ω6 alone showed a diminution in their performance in the Hole-Board test (Fig. 3). Comparative values recorded by healthy Wistar rats between 6th and 12th month of age remained normal. On the other hand, eSS rats showed a decline in their performance over time. At the end of 12th month, Control eSS untreated rats showed poorest performance.

### Histopathological analysis

eSS control animals did not show amyloid deposits in brain tissues or vascular lesions linked to atherosclerosis. Additionally, densitometric analysis of CT images showed areas of less homogeneity in eSS diabetic rat brains compared to Wistar rats. These alterations were less noticeable in animals that received ω6 + NDGA or ω3 + NDGA or even only ω3 (see Additional file 3).

**Fig. 4:**
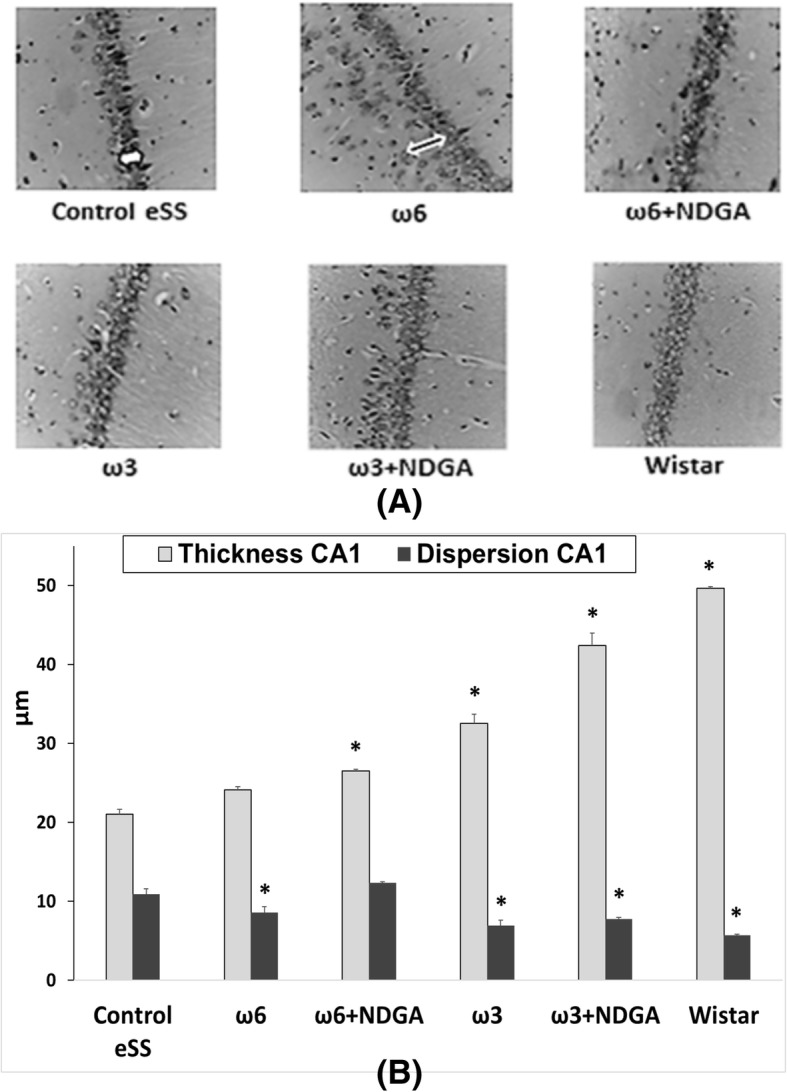
Histological sections of hippocampus. **a** Cell dispersion (black arrow) and thickness (white arrow) of the hippocampal CA1 zone, at 12 months of aging. Nissl 40x. **b** Histopathological analysis. Data are expressed as mean ± SEM * Indicate significant difference to Control eSS at the end of 12 months, *p* ≤ 0.05

Diabetic Control eSS, ω6 and ω6 + NDGA rats showed white areas in brain tissue images, which may be consistent with scattered spongiosis and gliosis, as well as greater thickness and cell dispersion of the hippocampal CA1 zone could be visualized (Fig. 4). The burden of these alterations was lower in eSS rats treated with ω3 alone or plus NDGA. These morphological abnormalities were not observed in healthy control Wistar rats.

### Apoptosis

**Fig. 5:**
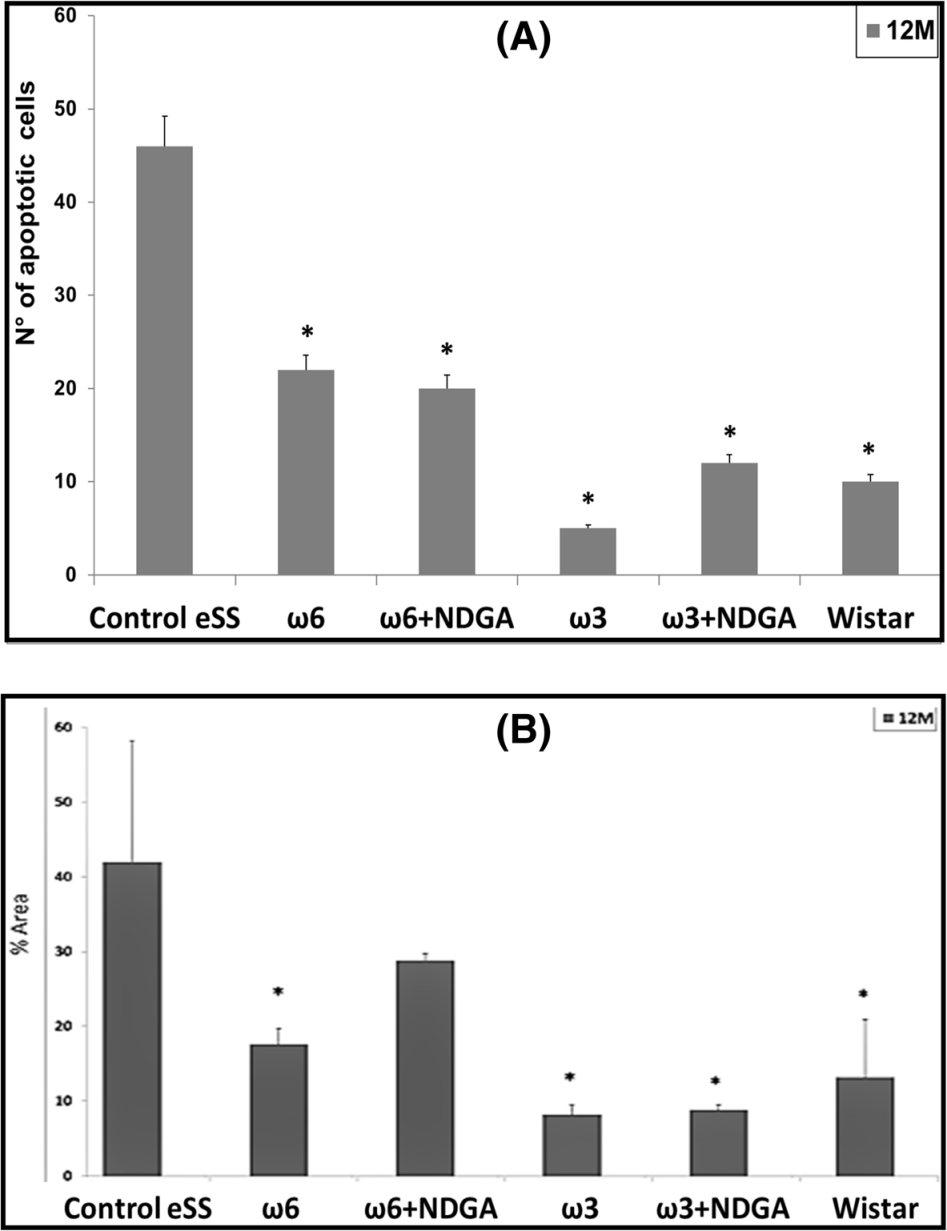
Apoptosis. Hippocampal apoptosis in animals at the end of 12 months (**a**) by flow cytometry, **b** by TUNEL. Data are expressed as mean ± SEM. * Indicate significant difference to Control eSS, *p* ≤ 0.05

Flow cytometry and TUNEL in dissected hippocampus of ω6 and ω6 + NDGA and eSS control rats showed significantly higher number of apoptosis than ω3 alone or ω3 + NDGA groups. Animals treated with ω3 PUFAs had the lowest number of hippocampal neuronal apoptosis (Fig. 5).

### Synaptophysin analysis

**Fig. 6:**
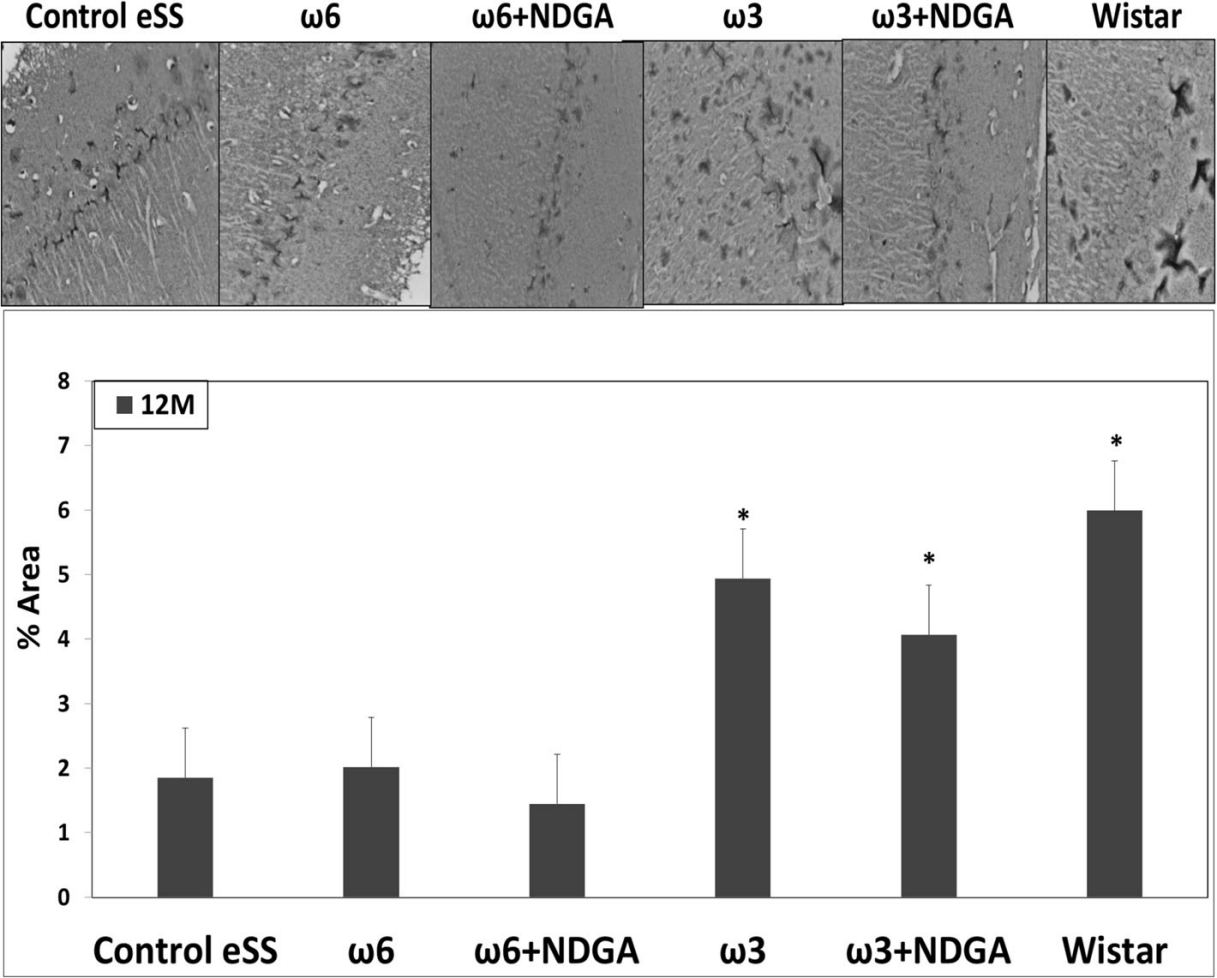
Immunohistochemical staining by synaptophysin. Analysis of levels of SYN protein in hippocampus slides. Data are expressed as mean ± SEM. *Indicate significant difference to Control eSS, *p* ≤ 0.05

Control eSS, ω6 alone or plus NDGA groups showed low immunostaining for SYN in hippocampus. Rats receiving ω3 or ω3 + NDGA showed levels of labelling like those observed for healthy Wistar rats (Fig. 6).

### Inflammation parameters

**Fig. 7:**
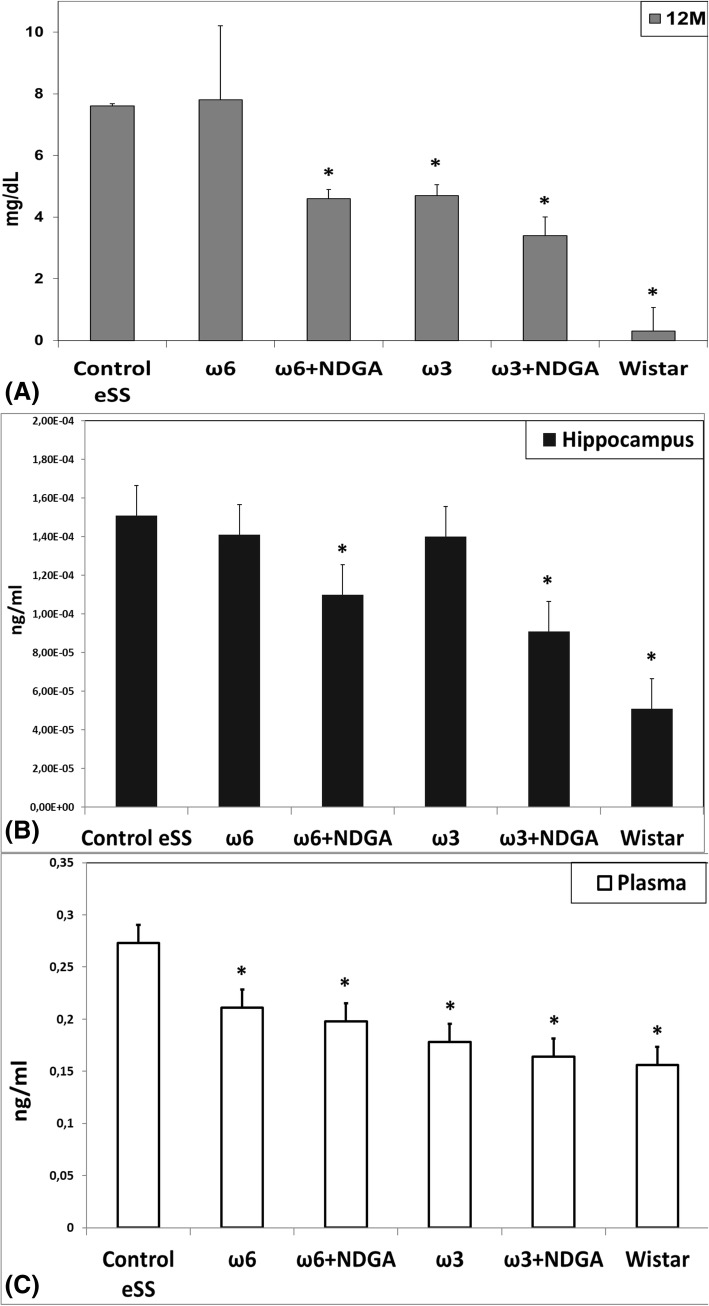
Levels of inflammatory markers. **a** Plasmatic high-sensitive CRP concentration. Levels of IL-6 measured in **b** plasma and **c** hippocampus. Markers were measured in animals at 12 month. Data are expressed as mean ± SEM. * Indicate significant difference to Control eSS, *p* ≤ 0.05

Plasma CRP levels were significantly higher (*p* < 0.05) in untreated eSS rats compared to healthy Wistar rats. Both groups of animals that received NDGA and those rats were treated with ω-3 alone, showed lower levels of CRP (Fig. 7). IL-6 values were significantly increased in plasma and hippocampus in Control eSS group. On the other hand, Wistar rats had the lowest levels of IL-6 values. Those animals that received treatment only with ω6 or ω3 showed an insignificant reduction, which became significantly decreased when NDGA was also administered (Fig. 7). In plasma, groups that were treated only with PUFAs or PUFAs plus NDGA showed a diminution of IL-6 levels and this was most evident in those that received ω3 PUFAs.

### Oxidative stress

#### Gamma-glutamyltranspeptidase activity

**Fig. 8:**
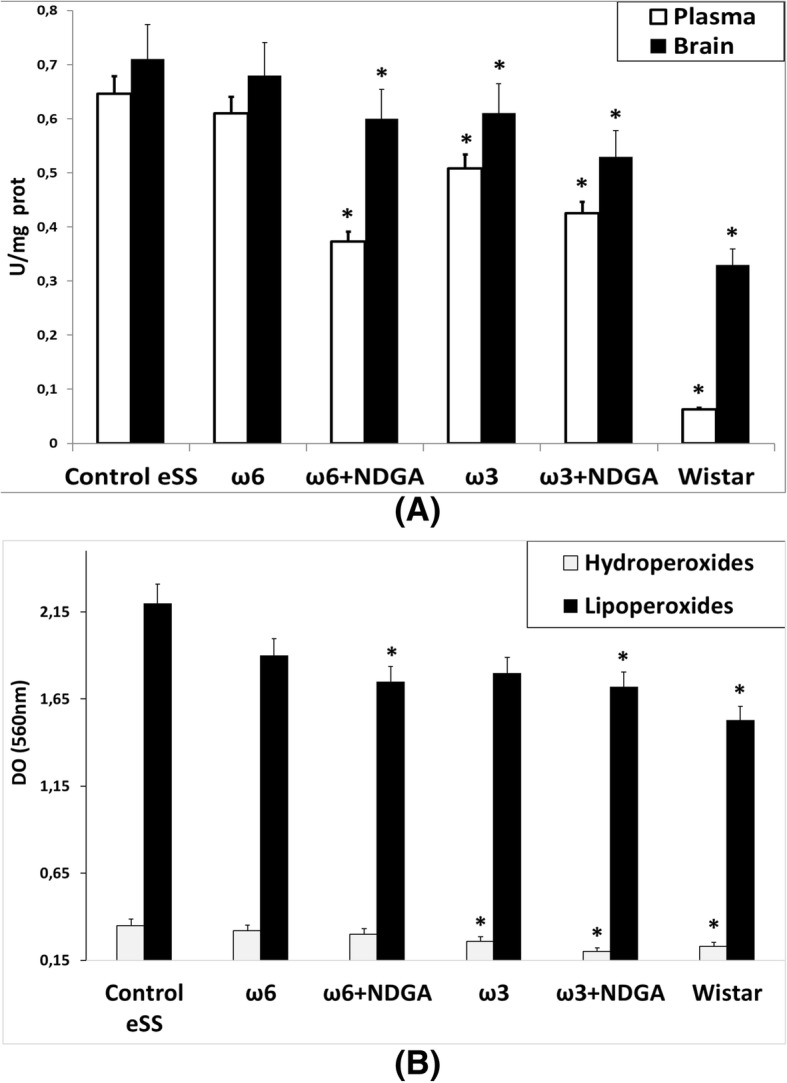
Stress oxidative markers. Markers were determined in animals at 12 month. **a** GGTP enzyme activity measured in plasma and brain tissue. **b** Plasmatic hydroperoxides and lipoperoxides assays. Data are expressed as mean ± SEM. * Indicate significant difference to Control eSS, *p* ≤ 0.05

Plasma and brain tissues of eSS rats showed higher levels of GGTP activity, a biomarker of oxidative stress. Rats that received NDGA or ω3 alone showed a significant decrease of plasma GGTP activity. ω6 alone treatment did not produce any significant effect on GGTP activity in the brain (Fig. 8a).

#### Peroxides

Higher levels of plasmatic peroxides (hydro- and lipo-) were recorded in control diabetic eSS and ω6 treated groups compared to healthy Wistar rats. Treatment with ω3 + NDGA decreased hydro- and lipo-peroxides levels in eSS rats. ω6 + NDGA treatment diminished plasmatic lipo-peroxides, whereas ω3 alone decreased only levels of hydroperoxides (Fig. 8b).

### Hepatic enzymes

To determine gross perturbations in the morpho-physiology of liver**,** plasmatic pyruvic oxaloacetic and glutamic oxaloacetic enzymes were assayed. Their levels were within normal values. Liver histopathological study showed neither steatosis nor any other hepatic lesion (results not shown). These data indicate that treatments employed in the present study have no effect on hepatic structure and function.

## Discussion

Behavioural performances, metabolic perturbations, images abnormalities and histopathological changes recorded in present work revealed that extensive brain damage compatible with DE is likely to occur in DM2 [4, 21, 22].

Profiles of plasmatic FA were modified in the experimental animals by treatments employed. The ratio of plasmatic ω6/ω3 was different among groups as shown in Table 2. It was higher in diabetic compared to Wistar animals. Interestingly ω6 group showed values no different from Control diabetic eSS rats. On the other hand, those animals administered with ω3 exhibited lower values of this ratio, similar to non-diabetic Wistar rats, which showed further decrease when NDGA was added. These findings suggest that low doses of fish oil when given for a long period of time produce desirable effects on PUFAs homeostasis as previously proposed [3, 23].

Activity of desaturases that are needed for fatty acid synthesis are known to be affected in DM2 animals and humans [24] that could explain differences in the levels of various PUFAs noted in the plasma among control, diabetic eSS and Wistar groups and improvements induced by low doses of ω3 PUFAs administration alone or in combination with NDGA.

DM2 is a chronic plurimetabolic disease whose mechanism of injury are associated with glucolipotoxicity [1, 4–7] an abnormal condition that is associated with increased activity of GGTP, hypertriglyceridemia, hyperglycaemia, insulin resistance and elevated levels of saturated free FA. All these metabolic abnormalities can, in turn, enhance apoptosis of pancreatic β-cells and neurons in the brain [4, 23–26]. PUFAs, especially ω3 have generally been considered to have a protective function in view of their anti-inflammatory and anti-apoptotic action [3, 4], as confirmed in the present study [3, 26–29]. Tarres and co-workers [10, 11] reported that eSS rats are a suitable model to study DM2 induced metabolic damage in view of the fact that these animals showed early development of hypertriglyceridemia and subsequently hyperglycaemia similar to what is observed in patients with DM2. eSS rats are not hypertensive and also did not show obesity or atheroma that could be related to neuronal damage [1, 3]. This implies that neuronal changes seen in this eSS diabetic model are solely due to metabolic changes induced by DM2.

Our results suggested that ω3 PUFAs ameliorate biochemical abnormalities associated with DM2 pathology [8]. The recorded plasma increment of MUFA in eSS rats compared to normal Wistar rats may be seen as a result of an attempt to elongate and desaturate precursor (18:1 ω9, OA) to long chained highly unsaturated FA, as previously discussed elsewhere [30, 31]. Out data reinforce the importance of certain dietary fats as potential risk factors associated with DM2 and its associated plurimetabolic nature.

At weaning, eSS were metabolically normal and even heavier than their normal Wistar counterpart (see Additional file 1). Only after weaning, they showed a progressive increase in glycaemia, OGTT and HbA1c values up to 6 months of age, values which remained elevated till the end of the study (Fig. 1). Since, by the end of 6th months of age eSS rats show an increase in TG, but not in Chol (Fig. 2), a phenomenon that could be related to glucolipotoxicity [3, 4, 25, 26] as previously observed by Tarres group [10, 11]. However, addition of ω3, ω6 PUFAs and NDGA treatments did not modify significantly these parameters.

Nevertheless, groups treated with ω3 alone or ω3 + NDGA showed improvements in the behavioural test, tomographic images, LGCI, oxidative stress and cell damage. These results could be related to a decrease in TG, CRP, IL-6, oxidative stress and apoptosis burden. Indeed, when cognitive behaviour was analysed by Hole-Board Test, eSS diabetics rats were more active than non-diabetic rats during the first weeks of age (Fig. 3), a finding previously described in diabetic mice by Toth et al. [20]. Hypermotility and hyperphagia may be linked to hunger for glucose thus triggering increased exploratory wandering. On the other hand, at the end of the experimental period decreased activity in all these three parameters: “Head dipping”, “Time Movement” and “Rearing” were different among the groups studied and Control eSS rats showed the most abnormalities (Fig. 3). Integrity of hippocampal nuclei is crucial for accurate control of rearing in challenging novel environments [15, 18]. Head dipping recruits and processes new spatial information [16], exploratory behaviour and neophiliac [14]. Normal moving is associated with a motor efficient system, especially with motivational-behavioural stimuli and others as functional plasticity processes which have origins in the dentate gyrus [17]. Our work showed that untreated eSS animals showed worse results in the elapsed experiment period, while the performance of animals treated with ω3 + NDGA recovered appropriate performance with marks similar to those obtained in healthy Wistar rats (Fig. 3). Hippocampus manages sensory afferents and synaptic mechanisms underlying certain types of fast learning as well as establishing crossed links to motivational, emotional, executive, and sensori-motor functions [32]. Experienced geriatricians reported that those suffering with long-standing hyperglycaemia in the form of DM2 show subtle changes in their usual behaviour. Many long standing DM2 patients exhibit difficulties in learning and progressive apathy, a pleomorphic scenario that becomes difficult to differentiate from depression or other neurodegenerative diseases [33]. Motor perturbations in patients are like those observed in eSS rats that are in agreement with data reported by Zhao et al. [34]. These researchers observed deficit of learning and memory in diabetic rats which they related to increased oxidative stress, endoplasmic reticulum stress and CNS cell apoptosis [34]. Whays et al., also observed altered behaviour in diabetic rats, concluding that a “depression-like” conditions and oxidative stress developed in chronic diabetic animals [35].

CT brain images showed in Control eSS rats and those treated with ω6 scattered patterns of heterogeneity (see Additional file 3) which resemble to those observed in long standing diabetics patients suffering from white matter lesions [20–22].

Thus, LGCI condition plays a key role as we proposed previously [3], since NDGA is an anti-inflammatory compound. Neuroimaging obtained in present work gives plausible anatomical comparative evidences similar to those observed in elderly DM2 patients, namely lacunar infarcts and cortical atrophy, giving further explanation for the cognitive declining seen in long standing diabetics patients  [20–22].

Histopathological abnormalities recorded in our work support described white matter and hippocampal lesions. Structural dispersion of neuronal layers, spongiosis, gliosis, cytoplasmic vacuolization, apoptosis and perikarion vacuolization- a known but not understood phenomenon described in DE were observed at hippocampus specially on the dentate gyrus and CA1 zones in eSS rats (Figs 4 and 5). These findings were previously described in type-1 and 2 diabetic rats by Amin [36]. Perhaps, it could be associated with the Golgi-ER axis stress, with their consequent “swelling” by increased synthesis and storage of abnormal glycoproteins as proposed by Zhao et al. [34]. The presence of abnormal cellular dispersion led us to consider damage to synapses associated with ED, as is seen in Alzheimer’s disease. Observed pyramidal layer CA1 zone thickness may be related to increased apoptosis. It is well known that synapse damage is a key progressive lesion in dementia [37]. SYN, a crucial presynaptic protein, also decreased in aged diabetic rats compared with age-matched controls [38]. SYN and synapses are affected in imbalanced neuronal metabolism in aged diabetic rats [39]. Coincidently immunostaining of SYN was less evident in our Control eSS as well as in animals treated with ω6, whereas animals administered with ω3 alone or plus NDGA, showed average labelling similar to Wistar rats (Fig. 6). So, synapse preservation maybe the results of the beneficial effects of the combination of ω3 and NDGA treatment in this model. Besides, CRP and IL-6 were elevated in brain and plasma in eSS groups (Fig. 7) giving support to observed structural changes and LGCI state. Taking all these evidences into consideration, it is supported that a pathophysiological association exists between LGCI and progressive brain damage seen in elderly DM2 patients [3, 40, 41]. Another pathophysiological feature that is verified in the present study which sustain the above conclusion was the elevated GGTP values recorded in plasma and brain of eSS rats (Fig. 8a). On the other hand, values of hepatic enzymes were normal suggesting that there is no hepatic damage that could account for high lipo- and hydro-peroxides values determined in our eSS rat model (Fig. 8b). These assays are reliable markers of cellular oxidative stress. Indeed, these are important and frequent mechanisms by which complications due to type 2 DM might occur [42], even in prediabetes state [43] as a result of metabolic dysfunction [44] that can cause DNA damage and neuronal apoptosis [45, 46]. Based on the results of the present study, it is tempting to suggest that exogenous supply of ω3 PUFAs may be a promising protective measure to prevent oxidative damage to CNS in elderly DM2 subjects [46–48].

DE pathophysiology is related to longstanding insulin resistance, glucolipotoxicity, LGCI and oxidative stress, which can trigger activation of AKT enzyme pathway [49]. The observed improvement in eSS rats by the addition of ω3 PUFAs may result in better insulin-sensitive mechanism either by an increase of IP3, the natural AKT activator and/or the ability of ω3 PUFAs, mainly DHA, to bind to nuclear peroxisome proliferator activated receptors (PPARα and PPARγ) that, in turn, improves learning and memory [49].

There is increasing evidence that pro-inflammatory cytokine-induced response, linked to LGCI state, is closely involved in the pathogenesis of DM2. Elevated circulating inflammatory markers such as CRP and IL-6 may precede the development of DM2 and associated complications [50, 51]. In our eSS rats, LGCI significantly increased with age. In addition, increased levels of IL-6 in plasma and hippocampus tissues was also noted (Fig. 7b and c). Interestingly, in eSS rats treated with ω3 PUFAs and NDGA showed a decrease in these indices [52, 53].

In summary we observed that in eSS diabetic rats a strong link exists among increased oxidative stress and LGCI together with histopathological abnormalities, and apoptosis that could be related to the clinical complications observed in DE in elderly DM2 subjects. Our results also suggest that administration of low doses of ω3 PUFAs and NDGA can protect against neuronal damage that occurs due to long standing DM2.

## Conclusions

Neurocognitive, behavioral, biochemical and morphological perturbations observed in this model of DM2 (eSS rats) are compatible with changes seen in DE. Our results support that pathophysiology of DE, which is associated with glucolipotoxicity, chronic inflammation and high oxidative stress markers, can be neutralized by ω3 plus NDGA administration leading to significant improvements from features of DE.

## Additional files

Additional file 1: Hole-Board test box. Illustration of the three behavioral patterns studied. (TIF 152 kb)

Additional file 2: Table of GLC data. Complete profiles of plasma total fatty acids of 12-month-old rats. (TIF 406 kb)

Additional file 3: Images of brain CT scans of rats under different experimental treatments and densitometric analysis of tissue homogeneity. (PPTX 361 kb)

## Abbreviations

AA: Arachidonic Acid
AD: Alzheimer’s disease
CNS: Central nervous system
CRP: C-Reactive Protein
CT: Computed tomography
DE: Diabetic encephalopathy
DHA: Docosahexaenoic acid
DM2: Type 2 diabetes mellitus
EPA: Eicosapentaenoic acid
ER: Endoplasmic reticulum
eSS: Stillman-Salgado rats
FA: Fatty acid
FAME: Fatty acids methyl ester
FFA: Free fatty acid
GC: Gas liquid chromatography
GGTP: Gamma glutamyltranspeptidase
HbA1c: Glycated hemoglobin
HE: Hematoxylin-eosin stain
IL: Interleukin
LGCI: Low grade chronic inflammation
NDGA: Nordihydroguaiaretic acid
OGTT: Oral Glucose Tolerance Test
PUFAs: Polyunsaturated fatty acids
SYN: Synaptophysin
TG: Triglycerides
