# Supplementary figures and images for: Diabetic encephalopathy: beneficial effects of supplementation with fatty acids ω3 and nordihydroguaiaretic acid in a spontaneous diabetes rat model
Source: Lipids Health Dis. 2019 Feb 8;18:43. doi: 10.1186/s12944-018-0938-7 (PMC6368734; doi:10.1186/s12944-018-0938-7)

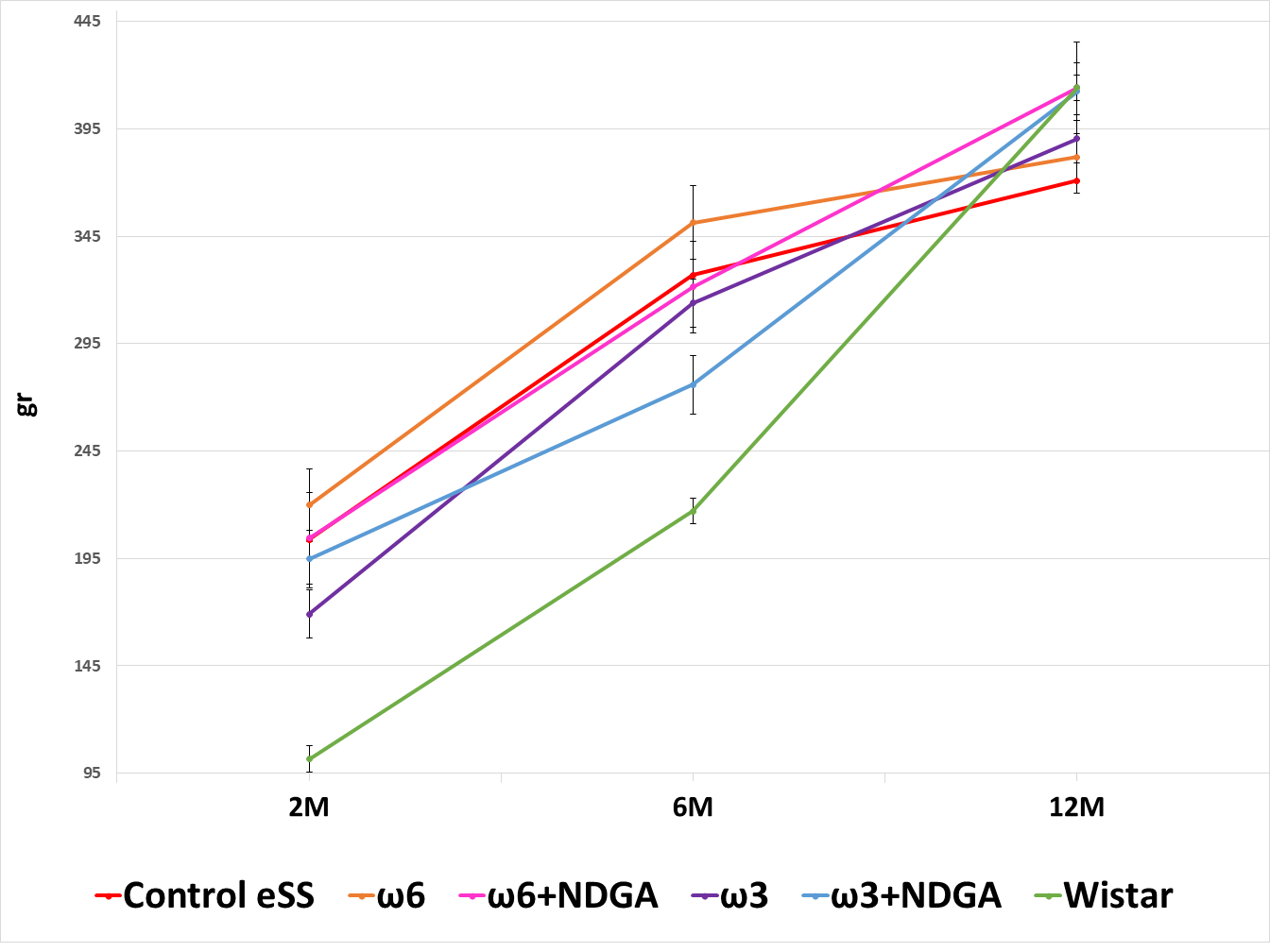

Supplement: Supplementary file 1 — Hole-Board test box. Illustration of the three behavioral patterns studied. (TIF 152 kb) [file 12944_2018_938_MOESM1_ESM.tif]

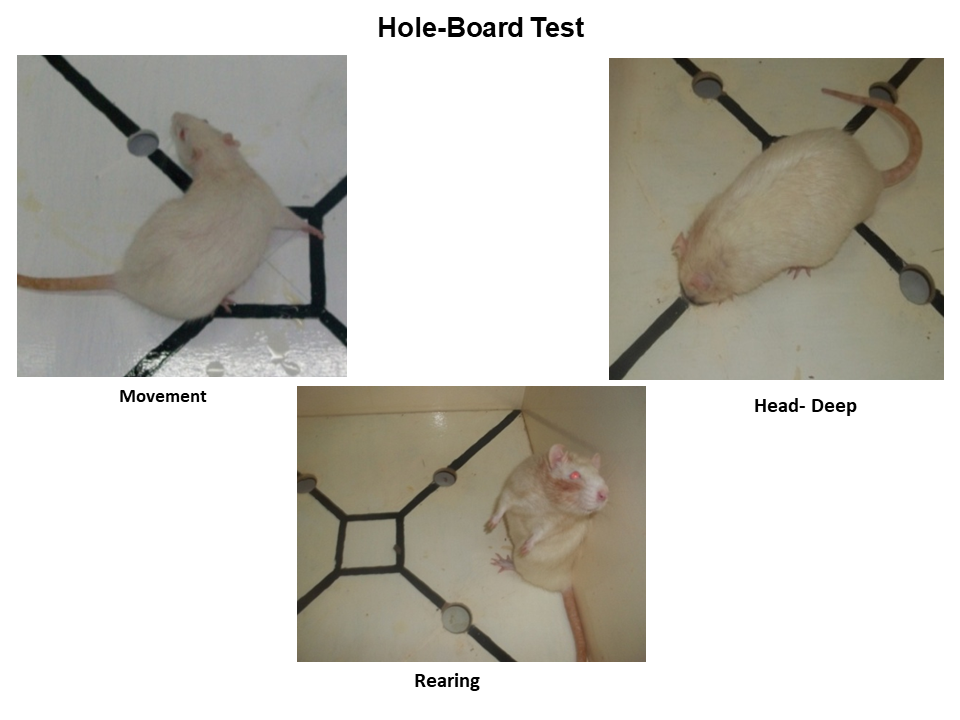

Supplement: Supplementary file 2 — Table of GLC data. Complete profiles of plasma total fatty acids of 12-month-old rats. (TIF 406 kb) [file 12944_2018_938_MOESM2_ESM.tif]
