# Supplementary material for: Diabetic encephalopathy: beneficial effects of supplementation with fatty acids ω3 and nordihydroguaiaretic acid in a spontaneous diabetes rat model
Source: Lipids Health Dis. 2019 Feb 8;18:43. doi: 10.1186/s12944-018-0938-7 (PMC6368734; doi:10.1186/s12944-018-0938-7)
Supplement: Supplementary file 3 — Images of brain CT scans of rats under different experimental treatments and densitometric analysis of tissue homogeneity. (PPTX 361 kb) [file 12944_2018_938_MOESM3_ESM.pptx]

## Slide 1
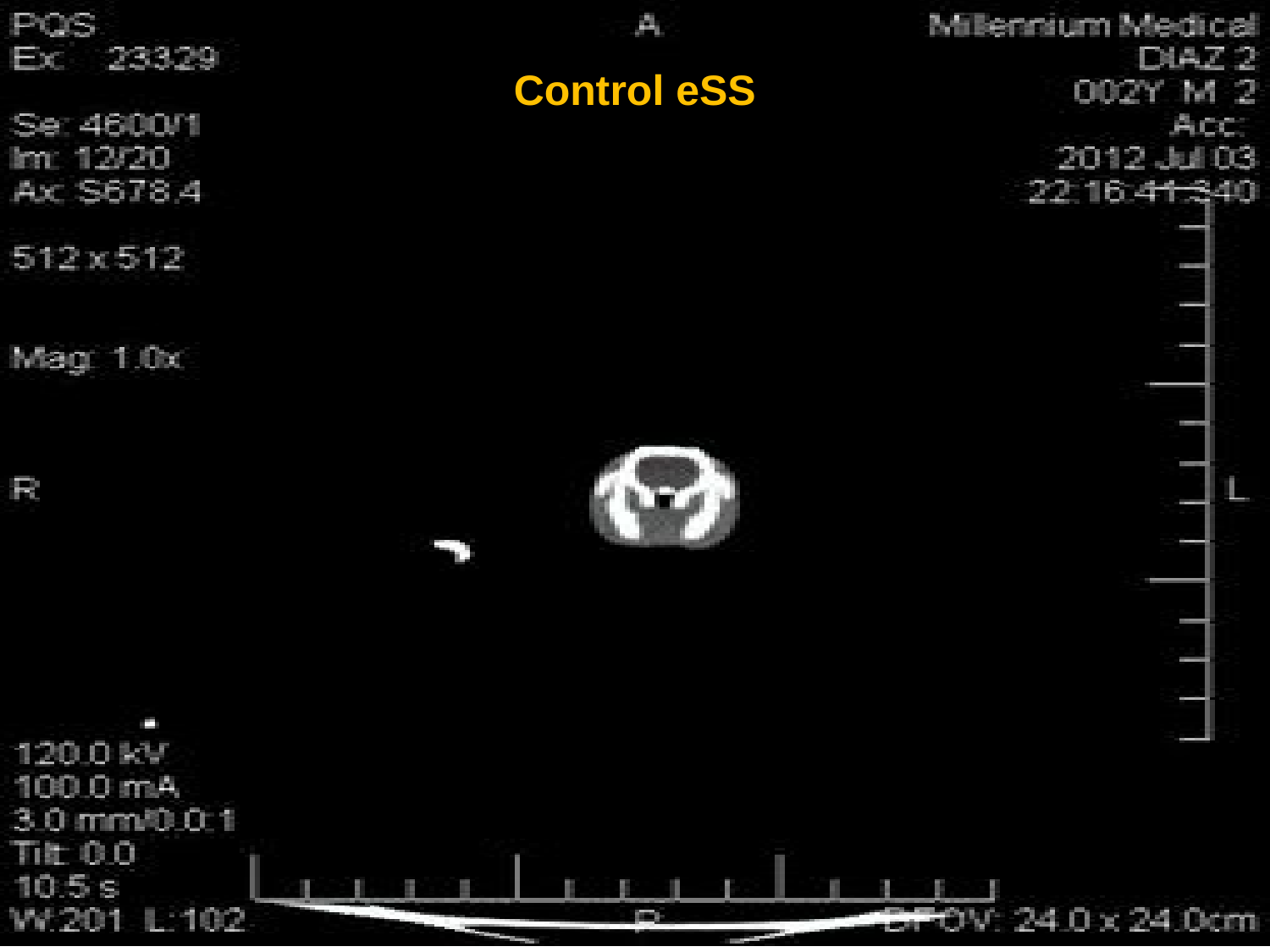

# C
Control eSS

## Slide 2
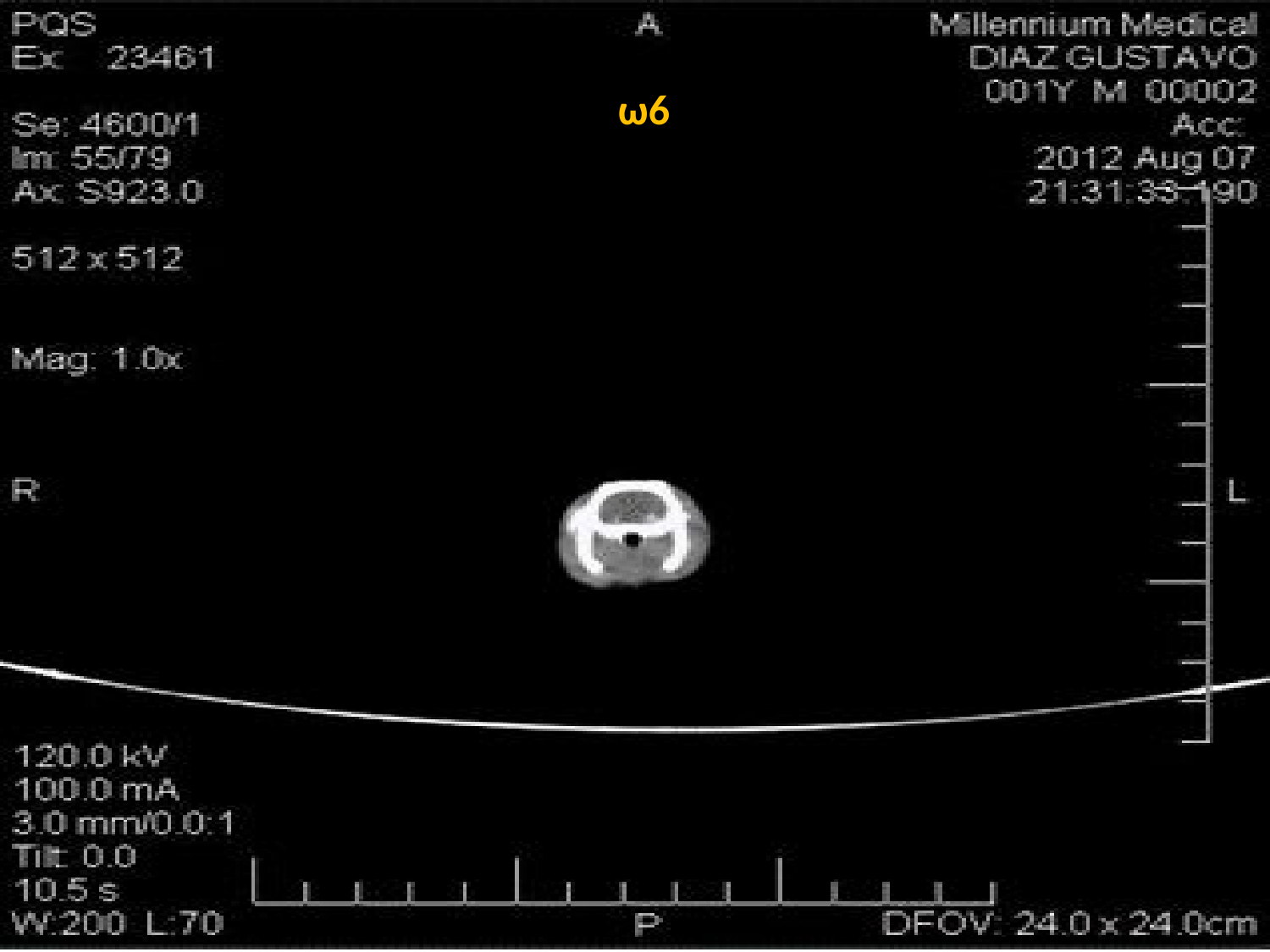

ω6

## Slide 3
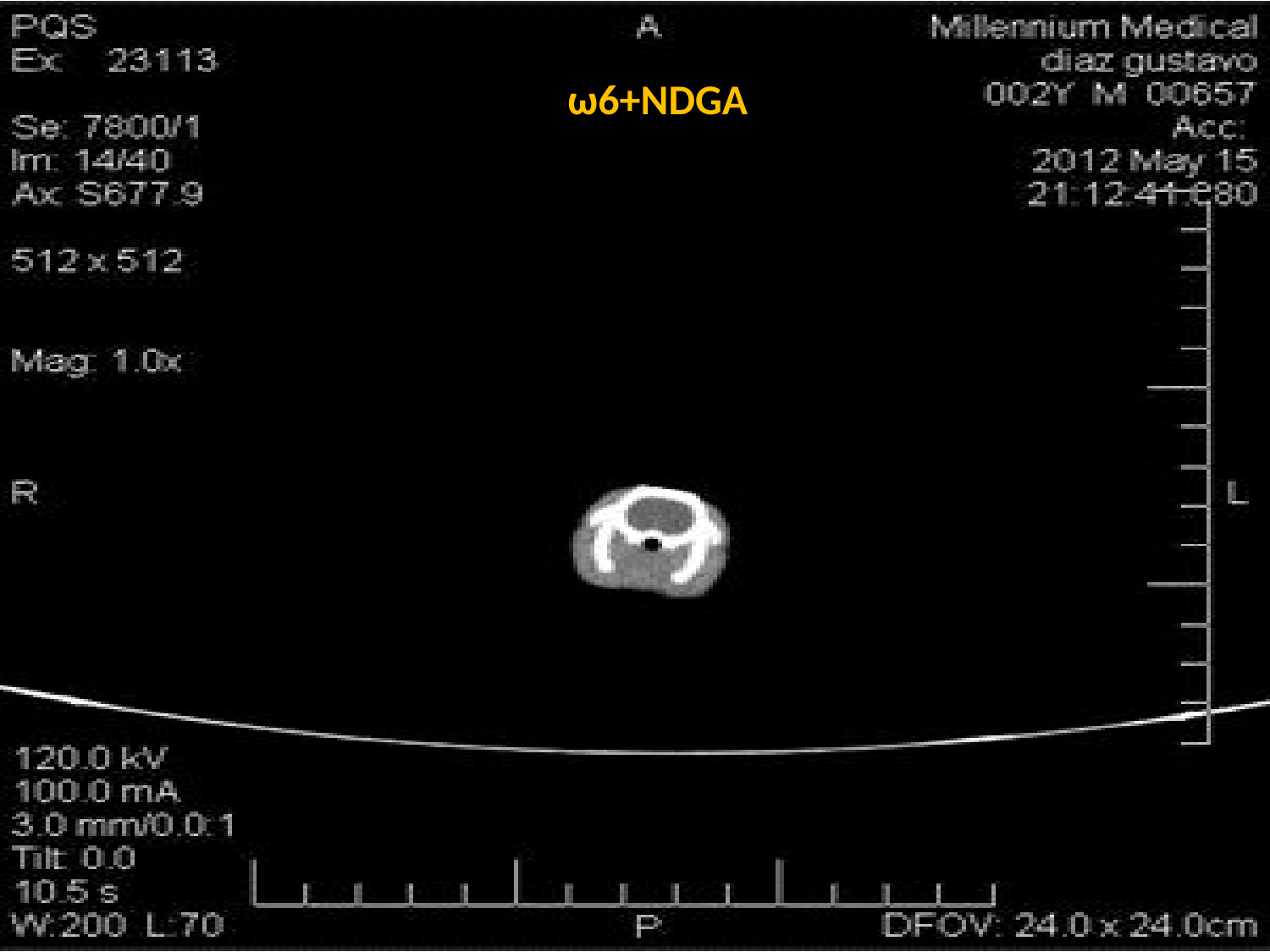

ω6+NDGA

## Slide 4
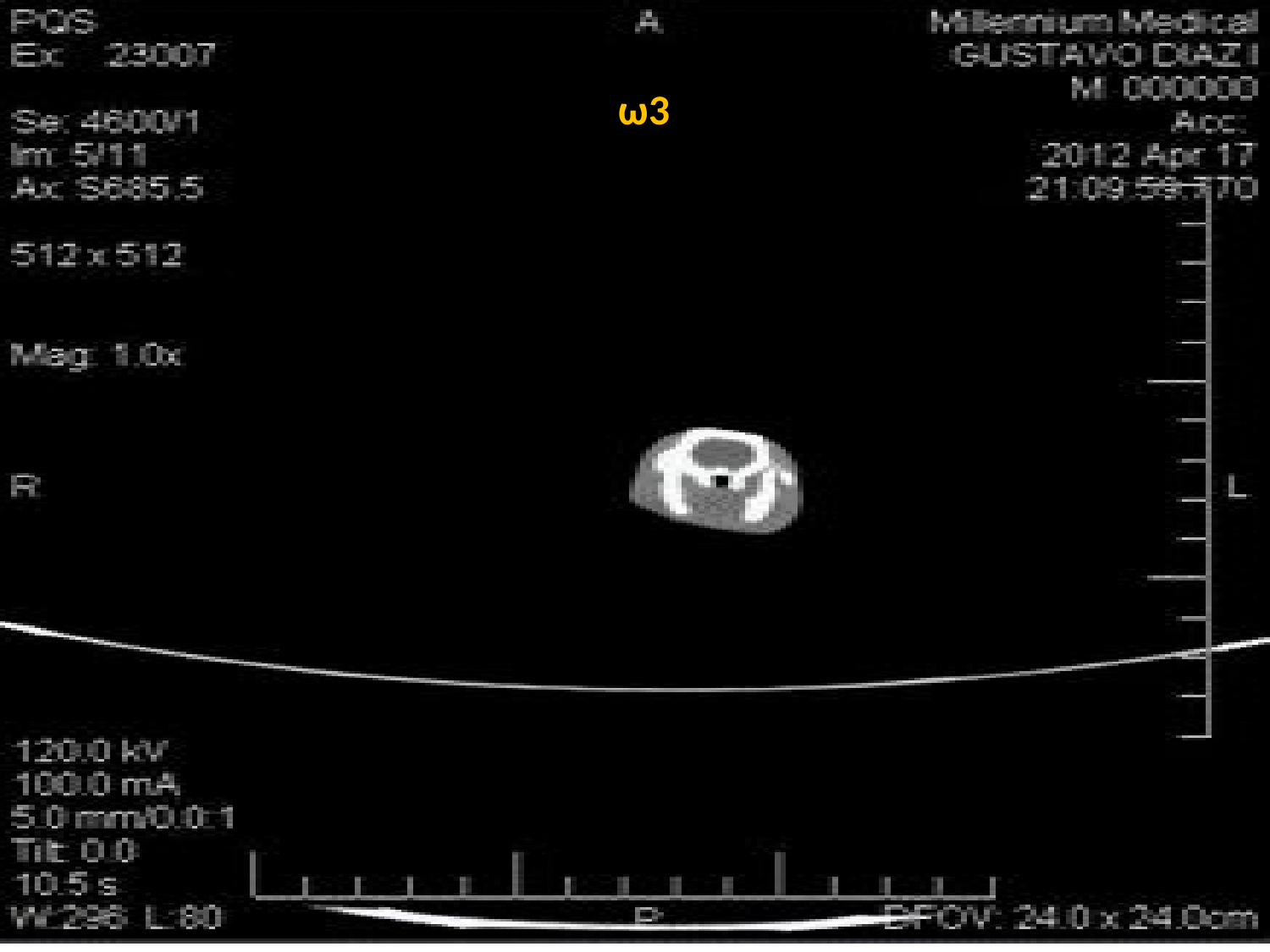

ω3

## Slide 5
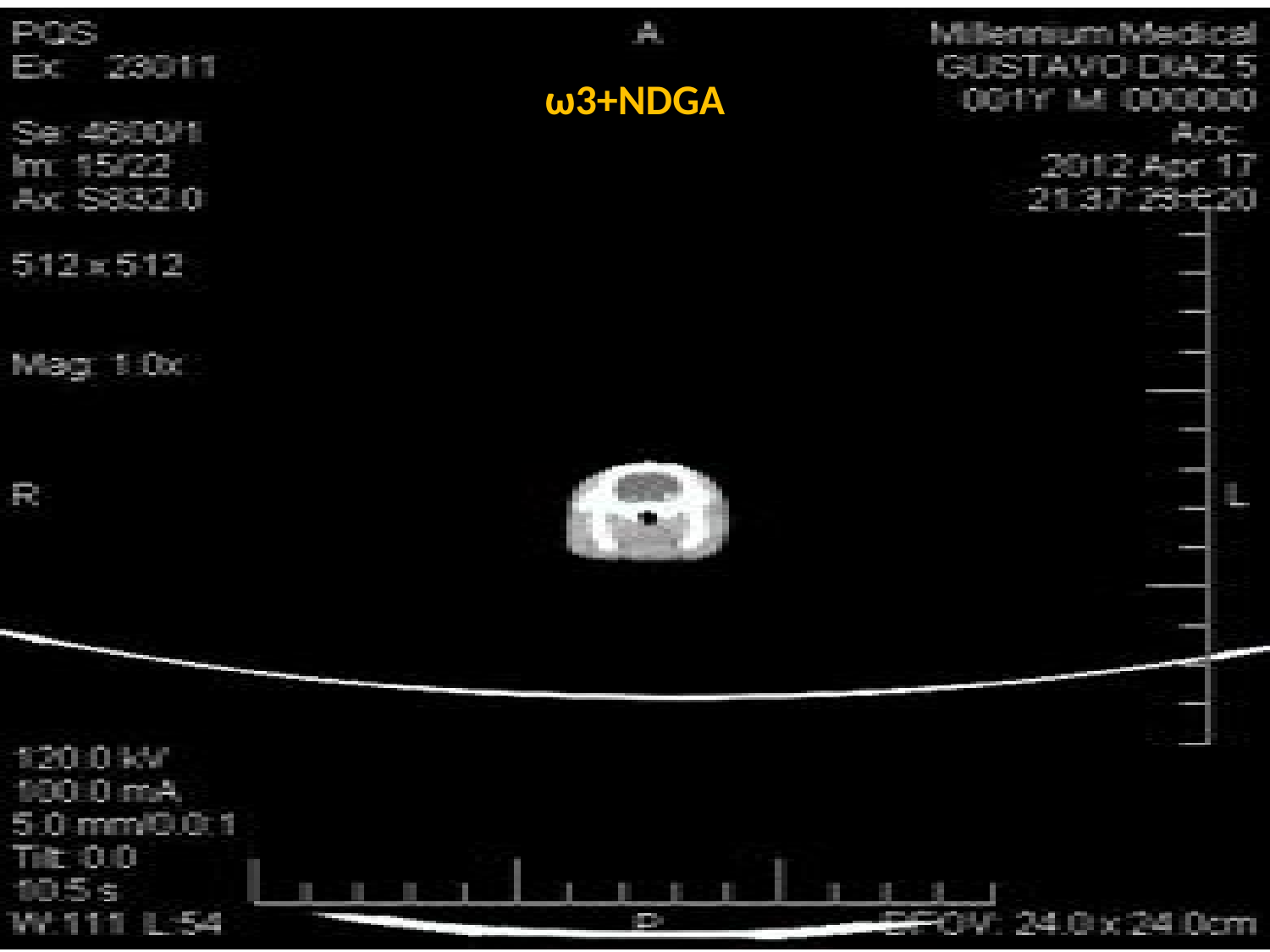

ω3+NDGA

## Slide 6
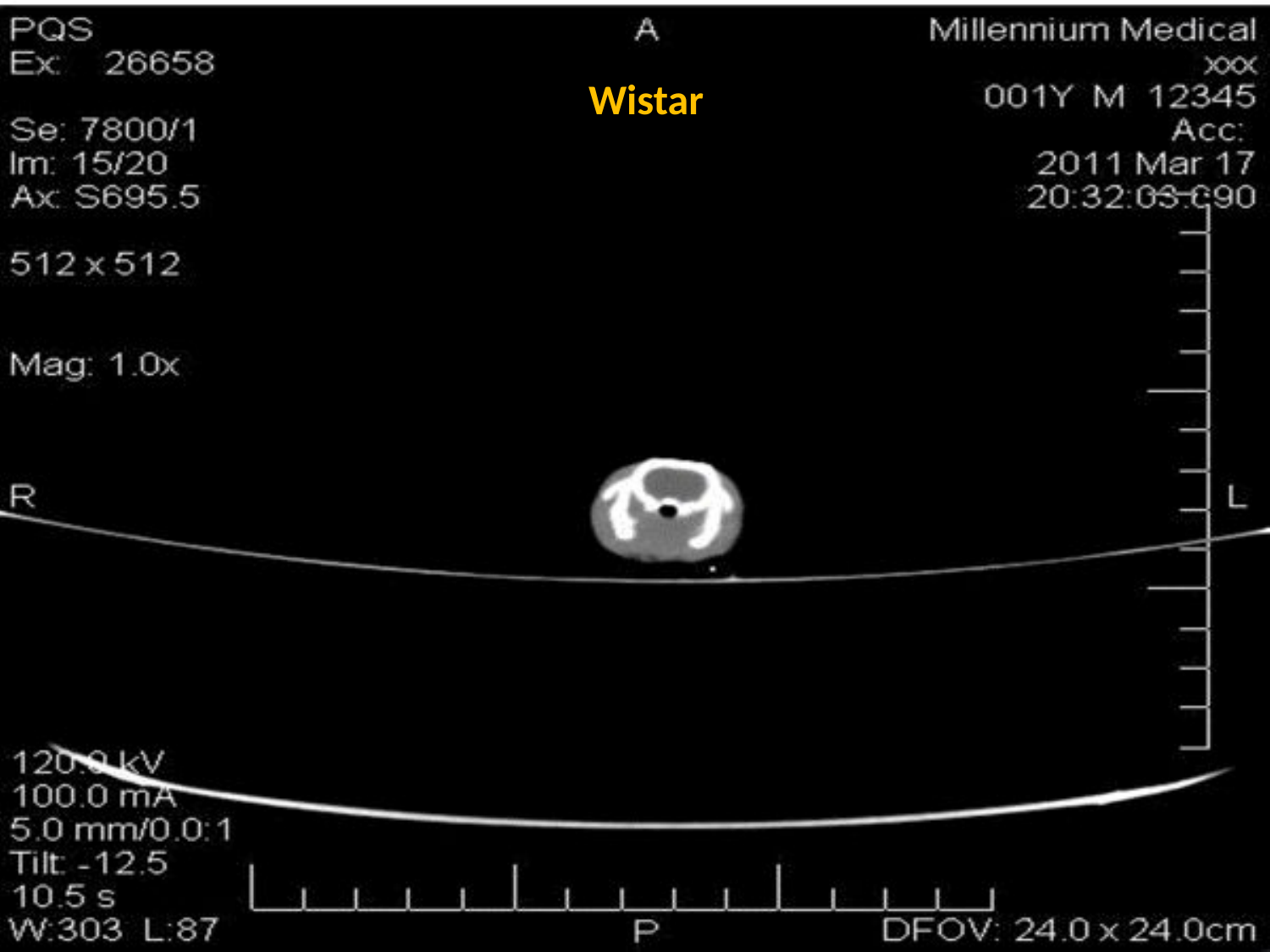

Wistar

## Slide 7
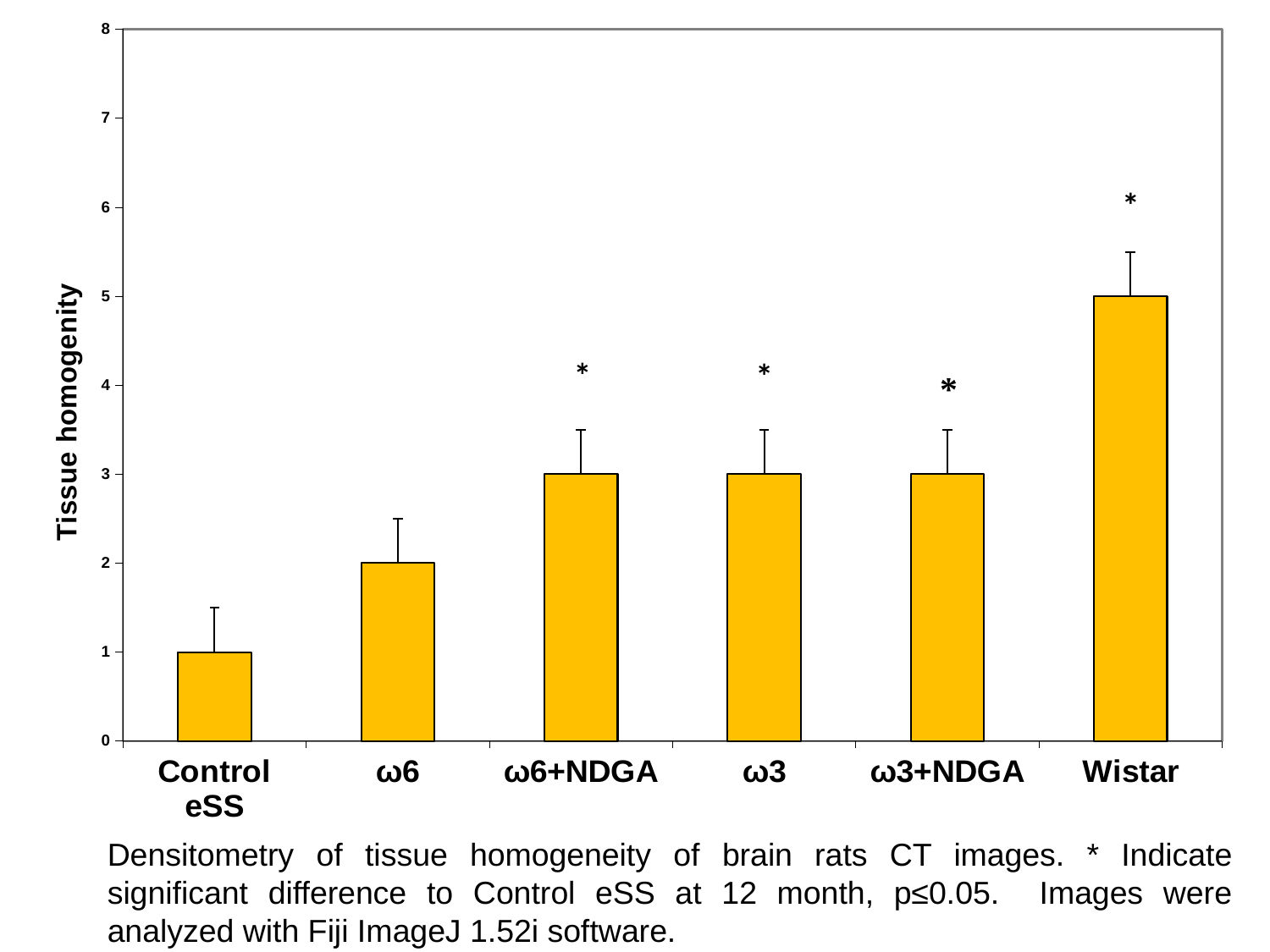

### Chart
| Category | 12M |
|---|---|
| Control eSS | 1.0 |
| ω6 | 2.0 |
| ω6+NDGA | 3.0 |
| ω3 | 3.0 |
| ω3+NDGA | 3.0 |
| Wistar | 5.0 |Densitometry of tissue homogeneity of brain rats CT images. * Indicate significant difference to Control eSS at 12 month, p≤0.05. Images were analyzed with Fiji ImageJ 1.52i software.
